# Single-cell analysis identifies Ifi27l2a as a novel gene regulator of microglial inflammation in the context of aging and stroke

**DOI:** 10.21203/rs.3.rs-2557290/v1

**Published:** 2023-02-15

**Authors:** Gab Seok Kim, Elisabeth Harmon, Manuel Gutierrez, Jessica Stephenson, Anjali Chauhan, Anik Banerjee, Zachary Wise, Andrea Doan, Ting Wu, Juneyoung Lee, Joo Eun Jung, Louise McCullough, Joshua Wythe, Sean Marrelli

**Affiliations:** The University of Texas Health Science Center at Houston; The University of Texas Health Science Center at Houston; Baylor College of Medicine; The University of Texas Health Science Center at Houston; University of Texas-Houston; University of Texas-Houston; The University of Texas Health Science Center at Houston; The University of Texas Health Science Center at Houston; The University of Texas Health Science Center at Houston; The University of Texas Health Science Center at Houston; University of Texas Health Science Center; McGovern Medical School/University of Texas Health Science Center at Houston; Baylor College of Medicine; The University of Texas McGovern Medical School at Houston, 77030, TX

## Abstract

Microglia are key mediators of inflammatory responses within the brain, as they regulate pro-inflammatory responses while also limiting neuroinflammation via reparative phagocytosis. Thus, identifying genes that modulate microglial function may reveal novel therapeutic interventions for promoting better outcomes in diseases featuring extensive inflammation, such as stroke. To facilitate identification of potential mediators of inflammation, we performed single-cell RNA sequencing of aged mouse brains following stroke and found that *Ifi27l2a* was significantly up-regulated, particularly in microglia. The increased *Ifi27l2a* expression was further validated in microglial culture, stroke models with microglial depletion, and human autopsy samples. Ifi27l2a is known to be induced by interferons for viral host defense, however the role of Ifi27l2a in neurodegeneration is unknown. *In vitro* studies in cultured microglia demonstrated that Ifi27l2a overexpression causes neuroinflammation via reactive oxygen species. Interestingly, hemizygous deletion of Ifi27l2a significantly reduced gliosis in the thalamus following stroke, while also reducing neuroinflammation, indicating Ifi27l2a gene dosage is a critical mediator of neuroinflammation in ischemic stroke. Collectively, this study demonstrates that a novel gene, Ifi27l2a, regulates microglial function and neuroinflammation in the aged brain and following stroke. These findings suggest that Ifi27l2a may be a novel target for conferring cerebral protection post-stroke.

Microglia (MG) are resident macrophages in the central nervous system (CNS). Critically, MG feature extensive heterogeneity, with subtypes evident across different regions of the brain, across different developmental stages and ages, and between the sexes^1,2^. These various MG subtypes play a pivotal role in both initiating, and resolving inflammation, and act in coordination with other glial cells such as astrocytes, and oligodendrocytes. The MG response to inflammatory challenge and ischemic stroke is a crucial component for maintaining/restoring brain homeostasis, salvaging tissue, and minimizing brain damage^3^. However, MG in the aged brain are less effective at exhibiting proper immune responses than MG in younger animals and instead exhibit uncontrolled inflammatory responses^4^. This loss of competence contributes to the impairment of brain function and facilitates aging processes in the aged brain^5,6^. Selectively restoring MG functionality in older animals so that they mirror a less pro-inflammatory, more anti-inflammatory-like state may be an effective intervention to mitigate ischemic damage and facilitate improved functional recovery after stroke in aged mice.

We performed scRNA-seq of young and aged brains of animals that underwent either sham surgery or permanent stroke. Using this unbiased approach, we discovered a gene, interferon *alpha-inducible protein 27 like 2A (Ifi27l2a*) that was mildly upregulated in MG of the aged brain and significantly upregulated in MG following stroke, and even more elevated in the aged stroke brains. To the date, however, no specific role for *Ifi27l2a* in ischemic stroke has been described.

Both interferons (IFN) and certain types of viral infection upregulate *Ifi27l2a* expression in the brain^7,8^. In non-glial cells Ifi27l2a protein enhances inflammation by blocking the action of nuclear receptors (NR4A) that normally act to promote expression of anti-inflammatory genes^9,10^. In the current study, we further demonstrate that reducing *Ifi27l2a* expression provides significant reduction of neuroinflammation and brain infarct following ischemic stroke. Together, these studies provide compelling new evidence that targeting Ifi27l2a expression or function may mitigate brain injury and inflammation following ischemic stroke.

## Results

### Ifi27l2a is highly upregulated after stroke in MG and aging significantly enhances this upregulation.

To define the transcriptional signature across multiple cell types in the post-stroke brain, we performed scRNA-seq of young (3-month-old) and aged (20-month-old) male and female C57BL/6J mice subjected to permanent distal middle cerebral artery occlusion (pdMCAO) or sham surgeries (Table 1). The pdMCAO stroke model was used since it produces both primary injury (cortical infarct) and secondary injury in the thalamus 2-weeks post-stroke^11^. Since the cortex and thalamus also feature clear increases in microgliosis and astrogliosis following stroke, we included a brain region containing both peri-infarct cortex and thalamus for scRNA-seq study^11^.

**Figure 1:**
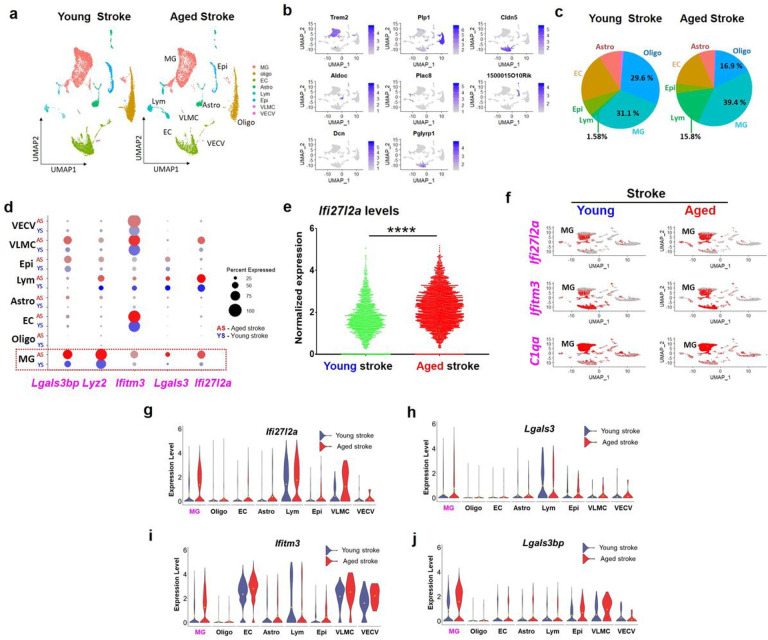
scRNA-seq identification of cell clusters and upregulation of *Ifi27l2a* in young and aged brains after stroke with distinct transcriptional signatures. **(a)** UMAP plot shows the clusters in young and aged stroke (Seurat *FindClusters* resolution at 0.02). MG; microglia, Oligo; oligodendrocytes, EC; endothelial cells, Astro; astrocytes, Lym; lymphocytes, Epi; epithelial cells, VLMC: vascular leptomeningeal cells, VECV; vascular endothelial cells, venous (**b**) Feature plot verifying clustering assignments by representative cell specific marker gene expression (Trem2; MG, Plp1; Oligo, Cldn5; EC, Aldoc; Astro, Plac8; Lym, 1500015l10Rik; Epi, Dcn; VLMC, Pglyrp1;VECV). (**c**) Pie plot showing the percentage of total for each cluster in young stroke and aged stroke. (**d**) *Lgals3bp, Lyz2*, *Ifitm3, Lgals3*, and *Ifi27l2a* were identified as the most highly expressed genes in aged stroke (red dot, aged stroke), compared to young stroke (blue dot, young stroke). Dot size indicates the percent of cells that express the respective gene in the cluster. (**e**) Normalized *Ifi27l2a* expression from total cell population in young stroke (12,708 cells) and aged stroke (8,165 cells). The overall expression of *Ifi27l2a* was greater in aged stroke. **** *p* <0.0001 unpaired *t*-test. (**f**) Feature plots showing the distribution of *Ifi27l2a, Ifitm3*, and *C1qa* in MG of young and aged stroke brains. Violin plots showed the increased expression of (**g**) *Ifi27l2a*, (**h**) *Lgals3*, (**i**) *Ifitm3* and (**j**) *Lgals3bp* on cell type-specific in young and aged stroke (showing increased *Ifi27l2a* expression in MG, Lym, and VLMC clusters in aged stroke samples).

We evaluated differential gene expression (DGE) patterns, as an initial approach for determining how the molecular signature of cells within the young and aged brain change post stroke. To measure the effect of aging in stroked brains, we first integrated young stroke (AGGR2) and aged stroke (AGGR4) data into a single analysis (Seurat package^12^) for analysis. Then, a single integrated analysis (data integration, PCA, UMAP and clustering, and DGE) was performed. Visualization of this merged dataset of 21,092 cells from the aged and young stroke mouse brain through dimension reduction by uniform manifold approximation and projection (UMAP) identified eight clusters of unique cell types based on gene expression differences. Identities were assigned to each of the eight clusters using the expression of conserved cell type markers, including microglia (MG, n = 7180) (*Trem2*), oligodendrocytes (Oligo, n = 5149) (*Plp1*), endothelial cells (EC, n = 3943) (*Cldn5*), astrocytes (Astro, n = 1698) (*Aldoc*), lymphocytes (Lym, n = 1495) (*Plac8*), epithelial cells (Epi, n = 1192) (*1500015O10Rik*), vascular leptomeningeal cells (VLMC, n = 127) (*Dcn*) and vascular endothelial cells, venous (VECV, n = 89) (*Pglyrp1*) (Fig. 1a–b). Notably, the proportion of MG and Lym clusters were highly increased in the aged stroke brain, whereas the oligodendrocytes were reduced (Fig. 1c). These findings are consistent with more extensive white matter injury in the aged stroke brain and correlate with increased MG-mediated neuroinflammation and lymphocyte infiltration. As we and others have demonstrated that MG are highly sensitive to inflammation and ischemic stress and act to regulate innate immunity in brains^3,13^, we focused our subsequent analysis on transcriptional changes within MG.

Interestingly, our unbiased analyses of 21,092 cells (combined from young and aged stroke brains) showed that *Ifi27l2a* was the most highly upregulated gene in MG clusters in aged stroke, compared to young stroke (Table 2). The top five genes that were significantly upregulated in MG in the aged stroke brain included MG related genes (*Lgals3, Lyz2, Lgals3bp*) and another interferon-stimulated gene (ISG), *Ifitm3*. Dot plots compared the expression level and percent of cells expressing the top five genes upregulated in aged stroke versus young stroke (Fig. 1d). Within the MG cluster, there was a notable increase in the percentage of cells expressing *Ifi27l2a* between aged stroke and young stroke (62.6% vs 29.3%) (Fig. 1d). Total normalized expression of *Ifi27l2a* from all cells showed increased *Ifi27l2a* expression in cells of aged stroke, compared to young stroke (Fig. 1e). *Ifi27l2a* was more highly upregulated in MG of aged stroke brain, suggesting aging may act synergistically with ischemic stroke to promote *Ifi27l2a* expression in MG (Fig. 1f). While most of the *Ifi27l2a*-expressing cells belonged to the MG cluster, *Ifi27l2a* expression was also detected in Lym and VLMC populations (Fig. 1g). The VLMC showed increased *Ifi27l2a* expression with aged stroke, whereas stroke-induced *Ifi27l2a* expression in Lym was not markedly altered by aging. Other MG markers, such as *Lgals3, Ifitm3* and *Lgals3bp*, were also upregulated in MG and other cells following stroke (Fig. 1h–j). In contrast, there was no synergistic effect of aging with stroke on *C1qa* expression, a MG marker gene (data not shown). As expected, a known marker of activated MG, Cst7, was increased in aged stroke compared to young stroke brain (Extended data Fig. 1a), confirming that MG were more highly activated in aged stroke brains than in young stroke brains. In addition, we found significant upregulation of *Apoe* and *Lyz2*, while *Aif1* level appeared only slightly increased in MG in aged stroke compared to young stroke (Extended Data Fig. 1b-d). Taken together, the scRNA-seq data suggest an age-dependent upregulation of *Ifi27l2a*, which occurs predominantly in MG after stroke.

### scRNA-seq revealed that aging itself is sufficient to increase Ifi27l2a transcripts in MG.

Following our finding that *Ifi27l2a* is upregulation following stroke in an age-dependent manner, we next sought to determine if aging alone impacts *Ifi27l2a* expression. Thus, we compared young and aged sham brains, integrating the young and aged sham operated samples (young sham - AGGR1, aged sham - AGGR3). Eight clusters were identified (Extended data Fig. 2a and 2b), including oligodendrocytes (Oligo) (*Plp1*), MG (*C1qa*), EC (*Cldn5*), astrocytes (Astro) (*Gpr37l1*), epithelial cells (Epi) (*Ttr*), lymphocytes (Lym) (*Nkg7*), vascular smooth muscle cells, arterial (VSMCA) (*Des*), and B cells (*CD79a*). To determine how aging affects the transcriptional landscape in MGs, we compared the expression and percent of cells expressing the previously identified top five MG genes (*Ifi27l2a, Lgals3, Ifitm3, Lyz2*, and *Lgals3bp*) in young and aged sham brains. All 5 genes that were upregulated in MG from aged stroke brain were also increased by aging alone (Extended Fig. 2c). Notably, *Ifi27l2a* transcript levels significantly increased with aging, as did *Rps27rt*. *C1qa*, on the other hand, was not dramatically altered between young and aged sham animals (Extended data Fig. 2d). We also confirmed the increased expression of *Ifi27l2a* in MG, Lym and B cell clusters in aged brains (Extended data Fig. 3a). The violin plots revealed modest upregulation of *Ifi27l2a* in MG in aged brain compared to young brains. Interestingly, we found significant age-dependent upregulation of ribosomal protein genes such as *Rpl35, Rps27rt*, and *Rps28*. This age-dependent upregulation of *Rps27rt* in all clusters, including MG and Lym (Extended data Fig. 3b), suggested aging-mediated changes in ribosomal complex composition in MG. Expression of two other genes associated with activated MG (*Aif1 and Il-1b*) were also modestly increased with aging (Extended data Fig. 3c-d). We repeated the same analyses comparing sham to stroke for aged (Extended data Fig. 4, 5, 6) and young cohorts (Extended data Fig. 7, 8). Together, these data suggest a synergistic effect of aging and stroke on *Ifi27l2a* expression.

Disease-associated microglia (DAM) are present in the aged brain, and significantly increased following stroke. DAM are a recently discovered sub-population of MG found in the brains of various neurodegenerative diseases, such as AD, PD and ALS (REFs). We asked if DAMs are increased in the aged stroke brain. A recent study reported that homeostatic genes, such as *C1qa, Ctss, Hexb*, and *Csf1r* are not upregulated during the transition of MG into DAM, whereas other MG related genes (*Spp1, Cst7, Lpl*, and *Itgax*) are highly upregulated in DAM^14^. To determine whether a DAM-like MG subpopulation is increased in the aged or stroke brain, we compared the number and relative percentage of DAM in sham and stroke. The DAM subtype was defined by the elevated expression of *Aif1, Spp1, Cst7*, and *Lpl* among all MG (filter applied: *Aif1* ^*high*^ and *Spp1* ^*high*^ and *Cst7* ^*high*^ and *Lpl* ^*high*^, threshold by count). As expected, we did not detect DAM (0.0%) in young sham brains. However, we a small number of DAM in aged sham samples (0.9%) (Extended data Fig. 9a) shows that stroke increased the percent of DAM in both the young and aged brain (10.2% in young stroke vs 17.9% in aged stroke). These data show that MGs are converted to DAMs during aging, but are DAMs are significantly increased following stroke.

Ifi27l2a is inversely correlated with DAM cell phenotype. We further analyzed our scRNA-seq data (aged sham and aged stroke) to determine whether *Ifi27l2a* plays a role in microglial activity, particularly in DAM. First, we examined whether *Ifi27l2a* expression correlated with expression of DAM-related markers, such as *Lpl, Spp1, Cst7* and *Itgax*. We subset MG into 4 different sub-clusters based on their levels of *Ifi27l2a* expression (normalized *Ifi27l2a* expression: 0.3–0.99, 1–1.99, 2–2.99, 3+). We consistently observed a negative correlation between *Ifi27l2a* and DAM related gene expression (*Lpl, Spp1, Cst7*) (Extended data Fig. 9b).

Furthermore, segregating MG into either an *Ifi27l2a* “high” or “low” expressing cells followed by correlation analysis with known DAM genes and MG homoeostatic genes revealed a negative correlation between *Ifi27l2a* expression and phagocytosis/DAM related genes (*Lpl*, *Spp1*, *Itgax*, *Cst7*, and *Tyrobp*), but not homeostatic genes or other MG genes (Extended data Fig. 9c). Comparing the expression of DAM genes and homeostatic genes between “low” and “high” Ifi27l2a MG subpopulations revealed that DAM-related transcripts were significantly reduced in *Ifi27l2a* “high” MG. However, homeostatic genes (*Aif1, C1qc, Hexb*, and *Gapdh*) were either not changed or slightly increased (Extended data Fig. 9c). Furthermore, MG genes which are down-regulated in DAMs (*Csfr1, Olfml3, Trem119*, and *P2ry13*) were increased in *Ifi27l2a* “high” MG (Extended data Fig. 9c). Together, these data show a strong negative correlation between *Ifi27l2a* expression and a mature DAM transcriptional signature in MG.

**Figure 2:**
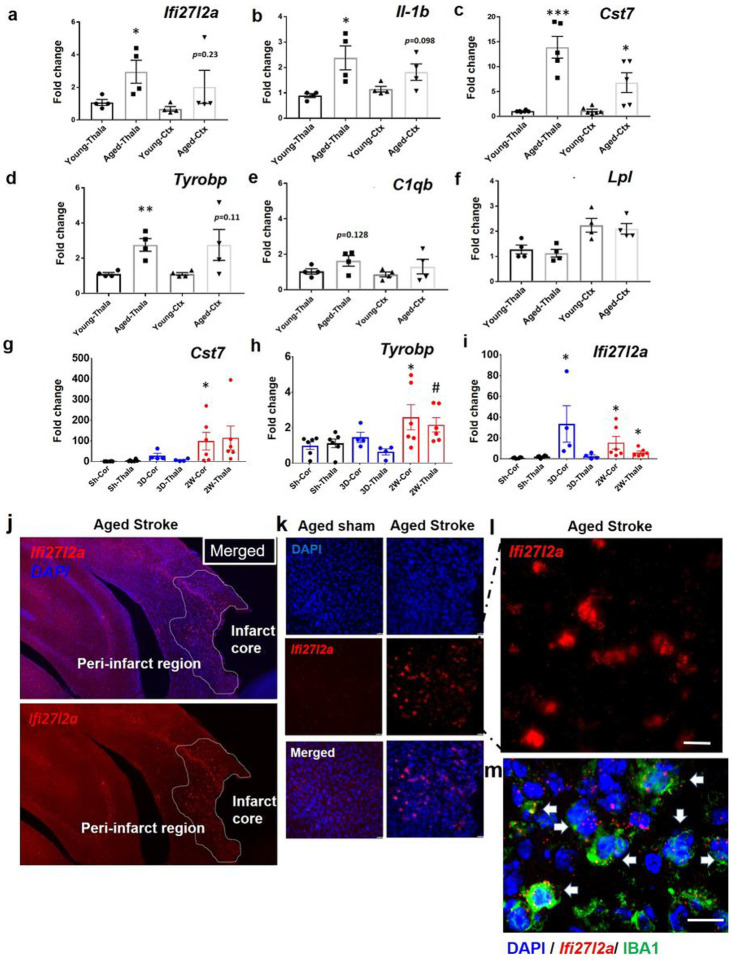
Regional increases of *Ifi27l2a* expression in brain with normal aging and in post-stoke brains. RNA was isolated from thalamus and cortex of young and aged brains for qRT-PCR analysis of (**a**) *Ifi27l2a* and other genes associated with MG phenotype: pro-inflammatory genes (**b**) *IL-1β*, (**c**) *Cst7* and phagocytosis related genes (**d**) *Tyrobp*, (**e**) *C1qb*, and (**f**) *Lpl*. Data presented as mean ± SEM (n = 4–6 mice per group). * p < 0.05, ** p < 0.01, *** p< 0.001 by two-tailed unpaired Student’s *t*-test. RNA was isolated from the cortex and thalamus of sham control or aged stroked mice at 3 days (3D) and 14 days (2W) after stroke for qRT-PCR analysis. Summary of fold change in expression for (**g)** *Cst7* (**h**) *Tyrobp*, (**i**) *Ifi27l2a*. Data presented as mean ± SEM (n = 4–6 mice per group). * p<0.05, compared to sham cortex (Sh-Cor) or Sham Thala (Sh-Thala), # <0.05, compared to 3D-Thala by two-tailed unpaired Student’s *t*-test. RNAscope assay shows regional and MG-specific expression of *Ifi27l2a* in aged brain after stroke. (**j**) A representative stitched image showing *Ifi27l2a* transcripts (red dots) in the peri-infarct area of aged brain at 2 weeks after stroke. (**k, i**) Higher magnification comparing *Ifi27l2a* expression in aged sham vs. aged stroke brain. Images representative of 4 mice, each group. Scale bar = 10 um. (**m**) Confocal imaging showing *Ifi27l2a* mRNA expression as well as Iba1 immunofluorescence (marker for activated MG) from the peri-infarct region. Examples of MG expressing *Ifi27l2a* are indicated by solid white arrows. Scale bar = 10 um.

Regional Ifi27l2a expression with natural aging. While scRNA-seq showed that *Ifi27l2a* transcripts are enriched in MG and other cell types (e.g. Lym, VLMC) in both young and aged stroke brains, we lacked any data on whether there was a regional basis for these changes within the brain (e.g. within the primary injury in the cortex or within the secondary injury region occurring within the thalamus). The cortex and thalamus were extracted from the brains of naïve young (3 months, n = 4) and aged male mice (18–20 months, n = 4) to determine if there were regional differences in *Ifi27l2a* expression. Notably, *Ifi27l2a* mRNA was significantly upregulated in the aged thalamus (Fig. 2a, p **< 0.05**). *Ifi27l2a* expression also approached upregulation in the cortex in aged brains (*p* = 0.23). These findings agree with our earlier scRNA-seq finding and suggest normal aging increases *Ifi27l2a* expression in the brain. In addition, MG related genes *Il-1b, Cst7*, and *Tyrobp* were markedly increased in either the thalamus or cortex of aged brains, further supporting an age-dependent increase in MG activation (Fig. 2b–d). Transcripts for *C1qb* and *Lpl*, two genes which are known to be associated with microglia phagocytosis, were indistinguishable between the two stages (Fig. 2e–f). These data indicate that *Ifi27l2a* is induced along with other genes associated with proinflammatory MG phenotype in the aged brain.

Regional and temporal expression of Ifi27l2a in aged stroke brain. To provide regional and temporal expression of *Ifi27l2a* and other MG-related genes following stroke, we analyzed young and aged thalamus and cortex by qRT-PCR at 3 and 14 days post-stroke. As expected, *Ifi27l2a* was significantly elevated at three days (cortex) and two weeks (cortex and thalamus) after stroke, compared to sham (Fig. 2i). We evaluated two other genes associated with MG activation (*Cst7*) and reparative phagocytosis (*Tyrobp*), and which were found to be elevated in our scRNA-seq analysis. Both *Cst7* and *Tyrobp* were increased in cortex and thalamus by two weeks post-stroke, but not by 3 days (Fig. 2g–h). These findings suggest that *Ifi27l2a* expression is associated with the earlier phase of MG activation following stroke. The delayed expression in thalamus reflects the slower progression of the secondary injury mechanism.

To provide spatial context to Ifi27l2a expression at the single-cell level, we profiled *Ifi27l2a* mRNA transcripts on mouse brain sections using single-molecule *in situ* hybridization (RNAscope). Probing for *Ifi27l2a* in aged sham and stroke brains revealed elevated transcripts in the peri-infarct area at 2 weeks post-stroke compared with sham-operated controls (Fig. 2j–l). Combining RNAscope for *Ifi27l2a* with immunostaining for Iba1 confirmed that the majority of *Ifi27l2a* transcript is present in activated MG in the peri-infarct region of the aged brain (Fig. 2m).

**Figure 3:**
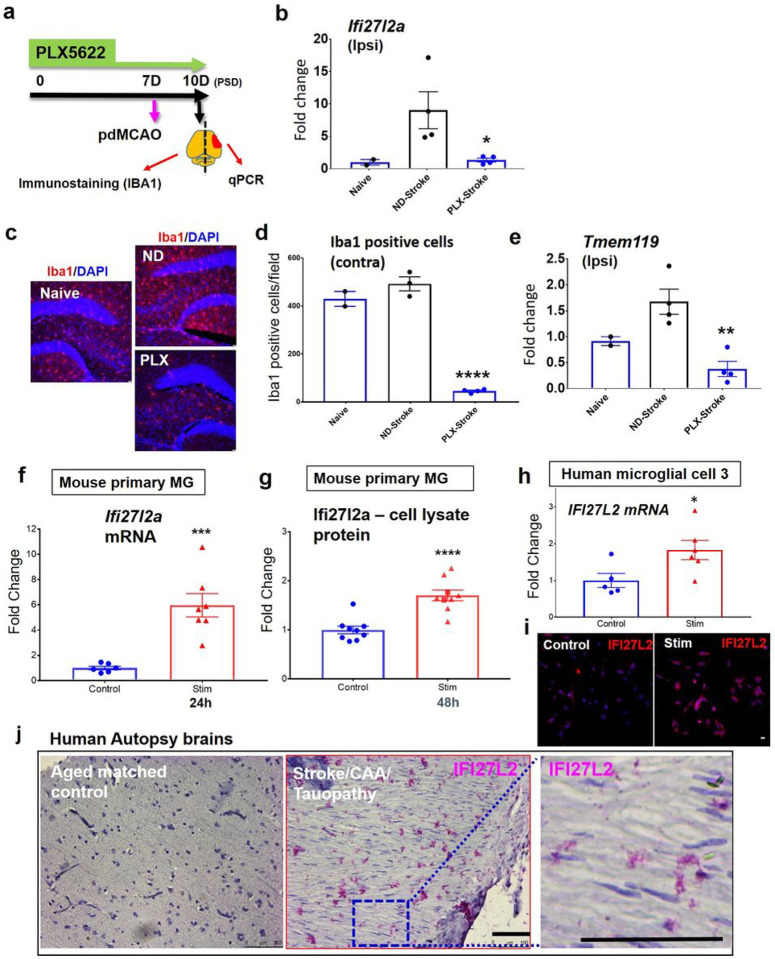
MG represent the primary source of *Ifi27l2a* upregulation following stroke. Depletion of brain MG with PLX5622 eliminates the stroke-induced increase of *Ifi27l2a* expression. Brain *Ifi27l2a* expression is reduced in PLX5622 treated mice after stroke. (**a**) Summary of experimental timeline and procedures. PLX5622 treatment is used to deplete the brain MG population. The brain hemisphere ipsilateral to the stroke was used for quantitative real-time PCR analysis, while the contralateral hemisphere was sliced for immunostaining to quantify MG depletion. Brains were evaluated at post-stroke day 3 (PSD 3). (**b**) *Ifi27l2a* expression was significantly reduced in PLX-treated mouse brains following stroke compared to ND-stroke. n=2, Naïve; n=3–4, PLX treated. (**c**) Representative images showing the significant reduction of Iba1 positive cells in PLX treated brains, compared to normal diet treated brains. (**d**) The number of Iba1 positive cells after stroke is significantly reduced by PLX5622, compared to normal diet (n=2, Naïve; n=3–4, PLX treated). Data presented as mean ± SEM. **** *p* < 0.0001 vs Naïve or ND-Stroke by one-way ANOVA with Bonferroni’s multiple comparison test. (**e**)Expression of *Tmem119* from the ipsilateral hemisphere of stroke mice with PLX5622 treatment (PLX) or normal diet (ND). Data represents mean ± SEM (n=2 naïve, n=4 PLX or ND). * *p* < 0.05, ** *p*<0.01 vs ND-Stroke by one-way ANOVA with Bonferroni’s multiple comparison test. (**f-j**) Upregulation of *Ifi27l2a*/IFI27L2 in stimulated primary MG, human MG and diseased human brain. Mouse primary MG were treated with TNFα (20ng/ml) and IFNγ for 24 hours (**f**) and 48 hours (**g**). *Ifi27l2a* mRNA were increased in stimulated MG for 24 hrs (n=6–7, *** p<0.001, two-tailed unpaired Student’s *t*-test). Intracellular Ifi27l2a protein was induced by proinflammatory cytokine treatment for 48 hrs (n=8–10, **** *p*<0.0001 two-tailed unpaired Student’s t-test). (**h**) HMC3 were treated with TNFα (20 ng/ml) and IFNγ (20 ng/ml) plus OGD (Stim). Induction of *IFI27L2* mRNA in stimulated HMC3 cells were assessed by qRT-PCR (n=5–6, * p<0.05, two-tailed unpaired Student’s *t*-test.). (**i**) Representative images show expression of IFI27L2 in stressed human HMC. Scale bar = 20 μm. (**j**) Human IFI27L2 protein expression in age-matched control human brain and stroke brain collected from patients with confirmed CAA pathology and tauopathy. IFI27L2 positive cells were found in the stroke/CAA/tauopathy brain samples (n=3, female), but not in controls (n=2, female). Scale bar =100 μm.

MG represent the predominant source of Ifi27l2a expression after stroke. Our analyses of stroked brains at post stroke day (PSD) 3 and PSD 14 revealed a significant increase in *Ifi27l2a* mRNA. To determine whether MG represented the predominant source for the increased *Ifi27l2a* expression, we used PLX5622 treatment to deplete MG in mice prior to inducing stroke. PLX5622 is a CSF1R antagonist that eliminates CNS-resident MG^15^. CSF1R mediated signaling is required for MG survival and proliferation^16,17^. Mice were treated with PLX5622 for seven days. On day 7 of administration of PLX5622, pdMCAO was performed. The PLX5622 diet was continued for 3 days after stroke surgery to prevent repopulation by MG. At PSD 3, brains were isolated and analyzed by qRT-PCR (ipsilateral hemisphere) and immunostaining (contralateral hemisphere). As a control, mice were fed normal diet (ND) for the same period (Fig. 3a). Notably, *Ifi27l2a* mRNA level was significantly reduced by 86% in PLX-stroked brains (ipsilateral hemisphere), compared to ND-stroked brains (Fig. 3b, p < 0.05). The effectiveness of PLX5622 to eliminate MG in brains was confirmed by Iba1 immunofluorescence (Fig. 3c–d) in the contralateral hemisphere. PLX5622 treatment resulted in a profound decrease in the number of MG in brains (Fig. 3d). Moreover, PLX5622 treatment significantly reduced *Tmem119* expression in brains after stroke, compared to naïve or normal diet administered brains (Fig. 3e, p < 0.05, compared to naïve and ND-stroke). These data indicate that the induction of *Ifi27l2a* after stroke is primarily dependent on the MG population in the brain.

MG induce Ifi27l2a/IFI27L2 expression with inflammatory stimuli. We next used cultured MG to evaluate the potential for inflammatory mediators to promote Ifi27l2a expression. First, we used mouse primary MG collected from the mixed glial cell culture obtained from P2 pups. Primary MG were treated with TNF-α (20 ng/mL) and IFN-γ (20 ng/mL) for 24 hours (to measure mRNA level of *Ifi27l2a*) and 48 hours (to measure protein level of Ifi27l2a by ELISA with cell lysate). Both mRNA (Fig. 3f) and protein levels (Fig. 3g) of Ifi27l2a were significantly increased with treatment.

To determine whether these findings extended to a human *in vitro* MG model, we challenged human microglial cells (HMC3) by addition of pro-inflammatory cytokines (TNF-α [20 ng/mL] and IFN-γ [20 ng/mL]) in combination with oxygen/glucose deprivation (inflammation/OGD). This inflammatory challenge induced a significant upregulation of *IFI27L2* mRNA in HMC3s (Fig. 3h, n = 5–6, p < 0.05). We found that human IFI27L2 protein level was dramatically induced at 20 hours post inflammation/OGD (Stim), compared to control treatment (Control) (Fig. 3i, representative of n = 4).

Given these results, we next tested if IFI27L2 protein was increased in the brains of patients that featured neuroinflammation. Sections from the brains of deceased patients without neurological disease (n = 2, female) and from stroke patients (n = 3, female) who also demonstrated cerebral amyloid angiopathy (CAA) pathology and tauopathy, in which neuroinflammation (microgliosis) is prevalent. Immunohistochemistry showed significant IFI27L2 expression in the stroke brain samples but low expression in age-matched control samples (Fig. 3j, representative of n = 2–3). Together, these data show the responsiveness of Ifi27l2a (murine) and IFI27L2 (human) to inflammatory stimulation and the presence of elevated IFI27L2 in brain of patients with multiple forms of neuroinflammatory disease.

Differential expression of Ifi27l2a in subtypes of microglia (MG) and macrophage (MΦ) populations in the aged brains following stroke. Given the extensive heterogeneity evident within MG and MΦ, we subjected the aged scRNA-seq datasets to more granular analysis to determine if *Ifi27l2a* expression profiles correlated with different functional roles. We ultimately identified a total of 28 clusters from brain cells of aged sham and aged stroke mouse brains, eight clusters of which were assigned an MG or monocyte/MΦ identity based on the expression of conserved cell markers (Extended data Fig. 10). Two MG homeostatic clusters were identified based on the expression of MG genes such as *Siglech*, *Tmem119, Gpr34, P2ry12*, and *Selplg*. These MG were annotated as *Siglech* homeostatic MG and *P2ry12* homeostatic MG. We also identified two different MG that appeared to be in an activated status (*Rag* + activated MG and *Tyrobp* + activated MG). Two Monocyte-Macrophage populations were also identified. We also found the disease-associated MG (DAM) like cluster showing high expression of *Lpl, Itgax, Cst7*, and *Spp1*. Note that these genes also correlate with the microglial genes and lipid metabolism genes upregulated in DAMs in other neurodegenerative diseases, such as AD^14,18^. Since we found that stroke and aging increase the expression of *Ifi27l2a* in MG, and that *Ifi27l2a* expression is negatively correlated with DAM genes, we asked whether expression levels and degrees of *Ifi27l2a* gene induction from sham to stroke in DAM would be different from MG in other sub-clusters. We therefore compared the degree of *Ifi27l2a* gene induction among MG sub-clusters in aged sham versus stroke brains (Table 3). Among the non-homeostatic MG clusters, *Ifi27l2a* induction in DAM (1.7 fold) is lower than any of the other activated MG.

### Ifi27l2a expression is sufficient to promote MG activation.

**Figure 4:**
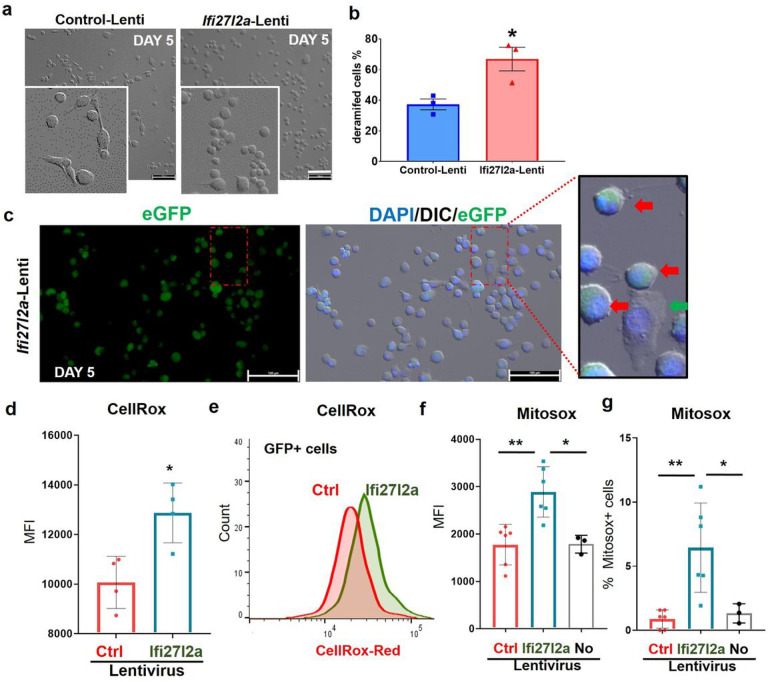
Ifi27l2a is sufficient for microglial activation and ROS generation. **(a**)Overexpression of Ifi27l2a by lentivirus changed the morphology at 5 days after infection. (**b**) The % of cells that changed their shapes was significantly increased in Ifi27l2a-lentivirus infected cells, compared to control-lentivirus infected cells (n=3, * p <0.05, two-tailed unpaired Student’s *t*-test). scale bar=100 μm (**c**) Representative images show that the cells that express Ifi27l2a (eGFP as an expression surrogate) changed their morphology to round and amoeboid shapes. Red arrows indicate cells that express Ifi27l2a and show a round morphology; Green arrow indicates a cell which does not express Ifi27l2a and remains in the intact morphology. scale bar=100 μm. (**d**) Ifi27l2a overexpression increased the intensity of CellRox dye in all cells (n=4, * *p*<0.05, two-tailed unpaired Student’s *t*-test). (**e**) Ifi27l2a overexpression increased CellRox intensity within GFP expressing cells (n=4, * p<0.05, two-tailed unpaired Student’s *t*-test). Mitochondrial ROS levels (**f**. MFI, **g**. % of MitoSox+ cells) detected by Mitosox was increased in Ifi27l2a lentivirus infected cells, compared to control (n=3–6, * p<0.05, ** p<0.01, one-way ANOVA with Bonferroni’s multiple comparison test).

Given the induction of *Ifi27l2a* in MG in aged brains and following stroke, we sought to elucidate the functional role of Ifi27l2a in MG-mediated neuroinflammation. Changes in microglial morphology is an early, quantifiable sign of inflammation in MG and MG functionality. Thus, we asked if Ifi27l2a expression alone (without additional inflammatory mediators) could induce a pro-inflammatory morphology in MG. We infected a murine microglial cell line (Sim-A9 cells) with a lentivirus where the Cx3cx1 promoter drove the expression of *Ifi27l2a* and an eGFP reporter, or a lenti-eGFP control. At 5 days post-infection, quantification of cell morphology showed that induction of Ifi27l2a expression caused an increase in the percentage of cells with a small, rounded shape (to a more amoeboid morphology or de-ramification) compared to lenti-eGFP control (Fig. 4a–b). Interestingly, MG with higher Ifi27l2a expression (using eGFP intensity as a surrogate maker) showed more dramatic morphological changes compared to cells that had low Ifi27l2a/eGFP expression (Fig. 4c). These results provide direct evidence that Ifi27l2a alone can initiate MG activation, even in basal conditions (i.e. inflammatory stimuli are not required).

### Ifi27l2a induces ROS production.

Earlier reports showed evidence for Ifi27l2a localization in mitochondria within non-CNS cells^19^. We also detected increased IFI27L2 in the peri-nuclear membrane and in mitochondria in HMC3 cells (not shown), leading us to question whether Ifi27l2a could mediate mitochondrial dysfunction in MG. Thus, we asked if Ifi27l2a expression alone could initiate the reactive oxygen species (ROS) generation in activated MG. We used CellROX Red and MitoSox. First, we utilized CellROX Red, a detector of most ROS species, to determine if Ifi27l2a expression induces ROS production in Sim-A9 cells in unstimulated conditions. Quantification by flow cytometry revealed that Ifi27l2a overexpression alone promotes a significant increase in ROS production (Fig. 4d, as expressed in median fluorescence intensity, MFI, Ctrl: lenti-eGFP control, Ifi27l2a: lenti-Ifi27l2a-eGFP, n = 4, * p < 0.05). Next, we only analyzed GFP positive cells, representing those with successful transduction. The ROS level was greater in Ifi27l2a expressing cells compared to eGFP only control cells (Fig. 4e, Ctrl: lenti-eGFP control, Ifi27l2a: lenti-Ifi27l2a-eGFP, n = 4, * p < 0.05).

We checked more specifically if mitochondria contribute as an Ifi27l2a-induced ROS source using Mitosox dye (specific indicator of mitochondria-derived ROS). Ifi27l2a overexpression resulted in a significant increase in mitochondria generated ROS level (Fig. 4f) and the percentage of Mitosox + cells (Fig. 4g). The “no-virus” cells (No) showed negligible effect on ROS levels. These data indicate that Ifi27l2a expression alone can cause ROS generation in mitochondria in activated MG, implying a causative role of Ifi27l2a in mitochondrial dysfunction in MG.

### Ifi27l2a hemizygous deletion is protective from ischemic brain injury in mice.

**Figure 5:**
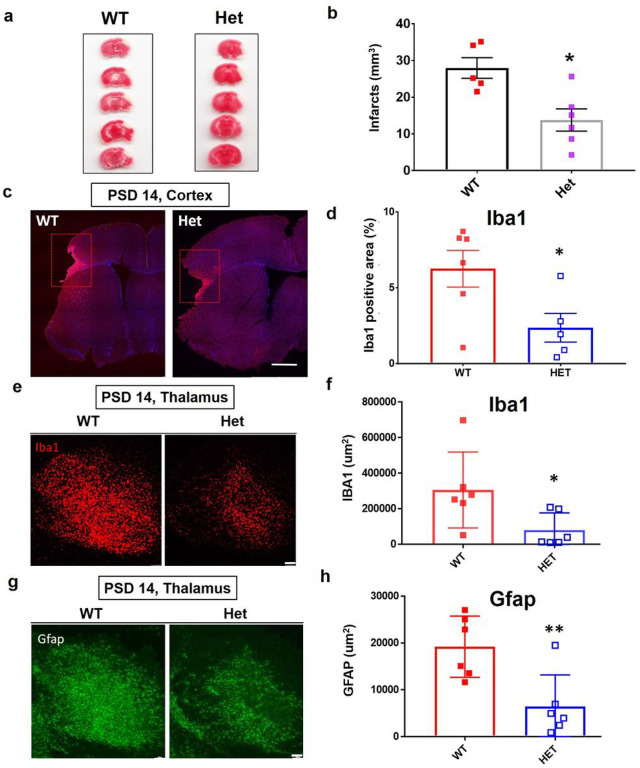
Hemizygous deletion of Ifi27l2a is neuroprotective for ischemic stroke. **(a, b**) Brain infarction at PSD 3 was significantly reduced in *Ifi27l2a*^+/−^ (Het) compared to WT (n=6, * *p*<0.05, two-tailed unpaired Student’s *t*-test). (**c, d**) Ifi27l2a deletion (Het) significantly reduced microgliosis in the peri-infarct cortex at 14 days following stroke (n=5–6, * p<0.05). Deletion of Ifi27l2a (Het) significantly reduced microgliosis (**e, f**) and astrogliosis (**g, h**) in the thalamus at 14 days post-stroke (n=6, * *p*<0.05, ** p<0.01, two-tailed unpaired Student’s *t*-test).

Given our finding that increased Ifi27l2a expression alone is sufficient to promote microglial activation, we asked if limiting Ifi27l2a expression could reduce microglial activation and brain injury following stroke. We used a permanent distal middle cerebral artery occlusion (pdMCAO) stroke model in WT and Ifi27l2a +/− (Het) mice (2–3 month old, male). At post-stroke day (PSD) 3, the infarct volume was significantly reduced in Het, compared to WT brain (Fig. 5a–b, n = 5 or 6, * p < 0.05). The area of activated MG (Iba1) was also reduced in the primary injury region at PSD 14 (Fig. 5c–d, n = 5 or 6, * p < 0.05). The pdMCAO model^20^ is also a well-established model for evaluating secondary injury in stroke; significant gliosis develops in the ipsilateral thalamus several days after the primary injury. We and others have shown significant gliosis in the ipsilateral thalamus 1 or 2 weeks following stroke^11,21,22^. Therefore, to evaluate the role of Ifi27l2a in secondary thalamic injury, we examined thalamic gliosis in WT and Het mice (2–3 months old, male) at PSD 14. Evaluation of the ipsilateral thalamus revealed significant reduction in both microgliosis (Fig. 5e–f, n = 6, * p < 0.05) and astrogliosis (Fig. 5g–h, n = 6 ** p < 0.01) in Het mice compared with WT. Note that the reduced injury in Het mice is not due to developmental differences in MCA territory. Analysis of vascular territory between WT and full *Ifi27l2a* KO revealed no difference (Extended Data Fig. 11, n = 6, p = 0.19). Together, these findings indicate that reducing Ifi27l2a expression can reduce primary and secondary injury associated with ischemic stroke, likely through attenuation of the microglial-mediated inflammatory response.

## Discussion

We used scRNA-seq to explore the effects of aging and stroke at the cellular level in the brain. As a result of these studies, we identified *Ifi27l2a* as a gene that demonstrated significant age-dependent upregulation in the post-stroke brain. This novel initial finding led to further study related specifically to where and when *Ifi27l2a* was upregulated in the brain and to the functional role of Ifi27l2a in aging, stroke, and other neurodegenerative conditions. From these studies, we now present the following major new findings: 1) *Ifi27l2a* is highly upregulated in MG following stroke, particularly in aged brain. 2) *Ifi27l2a* is mildly upregulated by aging alone in MG. 3) Upregulation of *Ifi27l2a* following stroke occurs predominantly in MG. 4) *Ifi27l2a* expression and upregulation following stroke varies by MG subtype. 5) *Ifi27l2a* expression is inversely correlated with gene markers of DAM cell phenotype. 6) Expression of *Ifi27l2a* alone promotes MG activation and mitochondrial ROS production. 7) Reducing Ifi27l2a expression provides reduced MG activation and ischemic injury in an ischemic stroke model. When considered as a whole, we now propose that inflammatory stress (caused by the aging process, ischemic stroke or other) initiates *Ifi27l2a* gene expression predominantly in MG, which then enhances and propagates inflammatory damage throughout the brain. Further, our data suggest that the level of Ifi27l2a expression in MG may serve as a molecular switch that triggers pro-inflammatory phenotypes and dampens reparative phenotypes in aging and following stroke. We discuss what is known about Ifi27l2a function and elaborate on our major findings below.

Interferons and interferon mediated signaling were originally identified as antiviral^23^, anti-proliferative and immunomodulatory mechanisms^24^ that were induced by viral infection. These pathways are most commonly known to play pivotal roles in host defense against viral infection. Accumulated evidence has also shown a critical role for interferon signaling (especially Type I IFN, α and β) in regulating neuroinflammation in aging and diseased brains, such as in the AD and stroke brain^25–28^.

However, how or if IFN signaling (e.g. Type I or Type II) modulates *Ifi27l2a* expression directly in stroke brain, especially in aged brain, has not been previously described. It has been shown that stimulation with IFNα and β, known activators of IFN type I signaling, can induce *Ifi27l2a* expression in cortical neurons^8^ and adipocytes^29^. However, it was unclear whether canonical Type I (α, β) or Type II (γ) IFNs could induce Ifi27l2a expression in microglia in a dish or in damaged brain to induce inflammation. Moreover, analysis of the *Ifi27l2a* promoter region (human ortholog, Isg12b) failed to show interferon-stimulated response elements (ISREs), which are thought to be required for interferon stimulated gene induction^29^. These findings suggested an alternative, non-canonical interferon-independent pathway for *Ifi27l2a* regulation, such as by microRNA or signaling via other foreign DNA/RNA sensing receptors. Indeed, our scRNA-seq data supports the notion of an interferon-independent pathway. We observed that while *Ifi27l2a* expression is markedly upregulated in MG, other representative Isg (*Mx1*, *Mx2*, Ifi family such as *Ifi27*, *Ifi35*, and *Ifnb1*, etc.) known to be upregulated by IFN response, especially type I Interferons (IFNα and β), were not measurably changed in MG. These findings suggested that another pathway (e.g. an IFN I independent pathway or combined signaling with IFN response and other intrinsic cellular signaling caused by ischemic or hypoxic insults) might be involved in the acute/chronic *Ifi27l2a* induction in MG. Such a scenario could be explained by the existence of other molecular hubs that relay the downstream signals to ultimately induce *Ifi27l2a* expression in MG after stroke. This possibility is supported by our scRNA-seq data showing significant upregulation of interferon regulatory factor 7 (IRF7) in MG from aged brain following stroke, whereas other IRFs were not markedly changed (data not shown). Moreover, in the series of *in vitro* experiments with primary MG treated with pro-inflammatory cytokines, a positive correlation was found between *Ifi27l2a* and *IRF7*, implying that IRF7 signaling may contribute to *Ifi27l2a* expression in activated MG. Interestingly, using TRANSFAC, a tool for transcriptional analysis, we found a putative IRF7 binding motif in the promoter of Ifi27l2a, suggesting that IRF7 may act as a key transcription factor to induce the *Ifi27l2a* gene expression in the inflammatory situation in microglia and other cells in brains. Further experiments will be required to specifically test the role and involvement of IRF7-mediated transcriptional regulation on *Ifi27l2a* expression.

Outside of the CNS, a limited number of reports have suggested a role for Ifi27l2a in facilitating inflammation though its interaction with other cellular partner proteins. During conditions of inflammation, it was shown that the Ifi27l2a protein is rapidly expressed and interacts with nuclear receptor 4A (NR4A) family members. The NR4A family is thought to support expression of multiple genes involved in attenuating inflammation in various kinds of cells^30–32^. Binding between Ifi27l2a and NR4A in the nucleus results in the export of NR4A to the cytosol, and thus the removal of a driver of anti-inflammatory and cytoprotective gene expression^10^. This model of NR4A regulation could also explain the novel role of Ifi27l2a in MG after stroke. In support of this possibility, a separate study showed that MG-specific *Nr4a1* knockout alone promoted inflammation and microglial activation as well as increased pathology in the experimental autoimmune encephalomyelitis mouse model (EAE)^33^. Interestingly, *Nr4a1* was also shown to play a critical role in maintaining an anti-inflammatory state of macrophages via attenuating NF-kB mediated pro-inflammatory gene expression^34^. Moreover, it was revealed that if *Nr4a1* is deleted in myeloid cells such as macrophages, more pro-inflammatory cytokines are produced^35^. It was recently shown that NR4A may also regulate phagocytosis via Mer tyrosine kinase (MerTK) gene expression in the cardiac repair process^36^. MerTK is a member of the MER/AXL/TYRO3 receptor kinase family, which is known to regulate phagocytic capacity in MG and MΦ. If other phagocytosis-related genes such as *Axl* and anti-inflammatory cytokines are also direct targets for NR4A, it is possible that lowering the expression level of Ifi27l2a might boost MG phagocytic capacity or promote phenotypical changes to DAM via promoting reparative phagocytosis related genes such as Axl. Indeed, our scRNA-seq data showed an inverse correlation between *Ifi27l2a* and *Axl*, indicating that lower *Ifi27l2a* expression may be a characteristic of the non-inflammatory MG phenotypes. We also found higher expression of *Nr4a1* in DAM and one of the homeostatic MG clusters. Overall, our data support the novel working model that Ifi27l2a regulation of Nr4a1 contributes to the phenotypic polarization of microglia in natural aging and in brain pathology. If our model is correct, the interaction of Ifi27l2a and Nr4a1 would represent a novel therapeutic target for reducing brain inflammation.

The other reported mechanism by which Ifi27l2a acts involves regulation of apoptosis. Studies in activated MG and other cells showed that Ifi27l2a can be shuttled to the mitochondria membrane, where it initiates a mitochondria-dependent apoptosis process^37,38^. Our study also supports this possibility wherein Ifi27l2a can act as an initiator for mitochondrial dysfunction by producing ROS. The mode of action of Ifi27l2a may also be regulated by its subcellular destination. With regard to the potential to trigger apoptosis and inflammation, we speculate that with more severe or prolonged MG activation, Ifi27l2a accumulation at the mitochondrial membrane might initially contribute to the MG inflammatory change, and then later trigger MG apoptosis. One intriguing hypothetical scenario is that high Ifi27l2a expression or mitochondrial targeting of Ifi27l2a contributes to the eventual termination of inflammatory MG. At this time, however, the potential role of Ifi27l2a in regulating apoptosis of MG after stroke has never been explored.

Our comparison of young and aged non-stroke brains (shams) showed an age-dependent upregulation of *Ifi27l2a* in MG, Epi, and B-cell populations in the brain. Expression of *Ifi27l2a* in these cell populations went from undetectable expression in young brain to moderate expression in aged brain. How aging promotes increased *Ifi27l2a* expression in these clusters is unknown. Since *Ifi27l2a* is among the known interferon stimulated genes (Isg) that can be upregulated by IFN-mediated pathways (viral infection or by inflammatory pathways^39,40^) chronic low-level activation of any of these pathways might promote the observed age-dependent increase in Ifi27l2a expression. However, given that the mice were housed in a specific pathogen free (SPF) facility, it is most likely that the increase we observed is due to low-level chronic inflammation that is known to exist in the aged brain^41,42^.

Expression of *Ifi27l2a* differed among the MG sub-clusters in the basal level of *Ifi27l2a* expression in aged sham brain and degree of *Ifi27l2a* upregulation following ischemic stroke. Expression levels of *Ifi27l2a* in sham brain were low in homeostatic subclusters and resulted in less upregulation in the post-stroke brain compared with activated MG subclusters. The activated subclusters showed 2.1–3.5 fold higher *Ifi27l2a* expression compared with homeostatic subclusters following stroke. The greater expression level in activated MG suggests a potential role for *Ifi27l2a* in microglial activation and proliferation. *Ifi27l2a* expression in two other MG subclusters is discussed further below. Our data showed an inverse correlation between *Ifi27l2a* expression and reparative MG genes, which are now recognized as DAM genes. This clear relationship in aged stoke brain suggests that Ifi27l2a could also be a key determinant for inducing DAM phagocytic activity or DAM phenotypical changes from non-activated or activated MG. It was further notable that the inverse correlation held up with groups of genes that are related to maintaining MG homeostasis and the DAM phenotype. These findings suggest that *Ifi27l2a* expression level may contribute to expression of genes in MG related to reparative and phagocytic function. Future study will be required to determine if the reduced *Ifi27l2a* expression is causative versus merely correlative for this MG phenotype.

In summary, using unsupervised scRNA-seq, we have found a significant increase in *Ifi27l2a* expression in MG following stroke, with particular upregulation in the aged stroke brain. Our data further show that mild *Ifi27l2a* upregulation even occurs with aging alone. We present evidence for a new model of MG phenotype regulation, wherein Ifi27l2a acts as a novel molecular regulator of microglial phenotypical changes and function. Based on the data we present here, we propose that elevated expression of Ifi27l2a contributes to a pro-inflammatory MG phenotype (producing more ROS and proinflammatory cytokines in MG) and reduced Ifi27l2a enables a non-inflammatory or phagocytic phenotype. Also we speculate that the functional role of Ifi27l2a is at least partly through negative regulation of Nr4a1-mediated gene transcription. In total, these findings suggest that targeting of *Ifi27l2a* expression or Ifi27l2a protein function in MG could be a novel strategy for regulating neuroinflammation in aging, stroke, or other neurodegenerative diseases to promote better functional recovery.

## Methods

### Animals.

All procedures were performed in accordance with NIH guidelines for the care and use of laboratory animals and were approved by the Institutional Animal care and use committee of the University of Texas Health Science Center. Sperm from Ifi27l2a^−/−^ KO mice [Ifi27l2atm1(KOMP)Vlcg] (REF) were obtained from the Diamond laboratory at Washington University in St. Louis and used for *in vitro* fertilization of WT (C57BL/6J) eggs (Genetically engineered rodent models core, Germ core, BCM). Resulting heterozygous Ifi27l2a^+/−^ progeny were backcrossed to establish the *Ifi27l2a*^−/−^ colony. Male and female mice in a C57BL/6J background (11–14 weeks old: young, 18–22 months old: aged) were used for all experiments. All animals were housed in the animal care facility at University of Texas Health Science Center and had *ad libitum* access to food and water and were maintained on a 12:12 light: dark schedule.

### Permanent distal middle cerebral artery occlusion (PDMCAO) model.

C57BL/6J mice of both sexes were used for scRNA-seq at 11–14 weeks or 18–22 months of age. PDMCAO was induced by permanent ligation of the right distal middle cerebral artery (MCA) using a micro-coagulator (Accu-temp)^11^. Mice were anesthetized with isoflurane (4% induction and 2% maintenance in airflow) and body temperature was maintained at 37°C by feedback-controlled heating pad and rectal temperature probe. Bupivacaine (0.25% at 1ml/kg) was injected subcutaneously (s.c.), prior to any skin incision^43^. The distal MCA was accessed via a craniotomy and permanently occluded just proximal to the anterior and posterior branches by electrocoagulation. Sham controls were generated with same procedure without electro-coagulation of the MCA.

For microglia depletion experiments, we used PLX5622, a CSF1R antagonist (REF). PLX5622 was provided by Plexxikon Inc. (Berkeley, CA) and formulated in AIN-76A standard chow at 1200 ppm by Research Diets Inc. PLX5622 was administrated for 7 days prior to the PDMCAO procedure and continued for 3 days after stroke. At 3 days after surgery, brains were isolated and ipsilateral hemisphere was used for RNA isolation and qRT-PCR analysis. The contralateral hemisphere was used for immunostaining.

### Brain sample preparation for single-cell RNA sequencing.

We processed brains from young and aged mice subjected to either sham or PDMCAO surgeries (Table 1). 14 days post PDMCAO (or sham surgery), Anesthetized mice were transcardially perfused with heparinized PBS (10 U/mL). Brains were removed from the skull and sliced coronally into 3 mm-thick blocks (from a region spanning + 1 to −2 mm from bregma), covering the cortical infarction and secondary thalamic injury site^11^. The brain slice was then minced with a razor blade and subjected to the brain tissue dissociation protocol (Miltenyi Biotec, Gladbach, Germany). Minced tissue was then incubated a collagenase/dispase mixture (150 μL of 1 mg/mL in 2 mL) for 30 minutes at 37°C in a gentleMACS Octo Dissociator (Miltenyi Biotec, Bergisch Gladbach, Germany) using the pre-installed program for adult brain dissociation. Myelin was removed using debris removal solution. Red blood cells were lysed and removed with red blood cell lysis solution. The final cell suspension was stained with trypan blue and live cells were counted using Countess II FL Automated Cell Counter (Thermo Fisher scientific, USA).

### GEM generation, library construction, and sequencing.

The 10X Genomics Chromium^™^, Single-Cell RNA-Seq System (10X Genomics, Pleasanton, CA) was used to prepare cells for scRNA-seq. Brain single cell suspensions were processed to generate barcoded cDNA libraries using GEM gel bead, Chip kit, and library kits (10X Genomics, Pleasanton, CA) as per the manufacturer’s instructions. Cells were partitioned with beads containing reagents (primers and RT) required for generating 10X barcoded cDNA in individual cell using Chromium^™^ controller. The resulting cDNA libraries were sequenced with NextSeq500/550 Hi Output Kit v2.5 (75 Cycles, 20024906) on an Illumina NextSeq 500 System.

### Sequencing data processing and analysis.

The cell ranger pipeline (10X genomics, Pleasanton, CA) was utilized to map the sequences to mouse reference genome (mm10), and to process barcode containing sequence data, aligning the read and generating feature barcode matrices that could be further processed by the Seurat package^12^ using R. We also used *cellranger aggr* pipeline to combine outputs from multiple samples into one output file. AAGR1 (5,706 total cells analyzed) was a combined population consisting of “sham brains of young male and young female” mice. AAGR3 (5,174 total cells analyzed) was a combined population consisting of “sham brains of aged male and aged female” mice. AAGR2 (12,866 total cells analyzed) was a combined population consisting of “Stroke brains from young male and young female” mice. AAGR4 (total 8,226 cells analyzed) was a combined population consisting of “Stroke brains from aged male and aged female”. Reads were mapped to the mm10 murine transcriptome (10X genomics, Pleasanton, CA). We used Seurat 3.1^44^ to analyze scRNA-seq data for clustering, and DEG identification between clusters and between the two groups. Briefly, log-normalization using *NormalizeData* was utilized. Feature counts for each cell were divided by total counts for the cell and multiplied by the scale factor (10,000). Then using log1p, data was natural log-transformed. The Uniform Manifold Approximation and Projection (UMAP) dimensional reduction technique (UMAP) was used for dimensional reduction and clustering was carried out using *FindNeighbors* and *FindClusters* with the resolution parameter either at 0.02 (generating 6–7 clusters) or at 1 (generating 25–27 clusters). Conserved cell type markers in each cluster were identified by using *FindConservedMarkers*. The name and level of genes that were differentially expressed in each cluster was determined using *FindMarkers*. Metadata and normalized read count data was extracted from Seurat objects and fed into Excel to further identify the critical genes (top 10 genes or top 50 genes) that were up- and down-regulated in each cluster and to find the correlation between levels of *Ifi27l2a* and other MG genes.

### Single molecule in situ hybridization (RNAscope).

The RNAscope fluorescent multiplex assay (Advanced Cell Diagnostics, Newark, CA, USA) was performed according to manufacturer’s instructions with 2 week post-stroke brains of aged mice (18–20 months) and aged sham to probe *Ifi27l2a* transcripts in brain cells. The murine *Ifi27l2a* probe was designed by ACD Biosystems, based on their own criteria. Brain sections (PFA fixed, 30-μm thickness) from the 2-week post stroke brain and sham brains of aged mice (18–20 month old) were hybridized with *Ifi27l2a* probes for 2 hours at 40°C. At the same time, ACD 3-plex positive control and negative control probes were incubated on one brain section to confirm signal specificity. The probes were amplified according to the manufacturer’s instructions and labeled with Opal-570 Red fluorophore (Akoya Biosciences, Marlborough, MA, USA). DAPI was used to label nuclei. Images were taken with a fluorescent microscope (Leica DMi8 fluorescence microscope system, Leica Biosystem, IL, USA) and a confocal microscope (Leica TCS SPE confocal system, Leica Biosystem, IL, USA). Multiple images were captured with the 10X objective covering the hemisphere and stitched to generate a single image (Leica LAS X software).

### HMC3 cell culture.

Human microglial cell line 3 (HMC3) cells were purchased from ATCC (CRL-3304, USA) and cultured in Dulbecco’s Modified Eagle’s Medium (DMEM) (Thermo Fisher Scientific, Waltham, MA, USA) containing 10% fetal bovine serum, 20 ng/mL recombinant human M-CSF1 (Tonbo Biosciences, San Diego, CA, USA) and antibiotics (Pen/Step) in 5% CO_2_ and 37°C.

### Brain processing and immunostaining.

For detecting Iba1 in PLX- or normal diet-treated mouse brains and gliosis (Iba1 and Gfap) in the thalamus following stroke, we performed immunostaining as previously described^11^. Cardiac perfusion with PBS, followed by 4% PFA (paraformaldehyde in PBS) were performed to clear the blood in brains. Perfused brains were then submerged in 30% sucrose in PBS for 24 hours at 4°C prior to sectioning at 30 μm thickness (Micron HM 450, Thermo Fisher Scientific, Waltham, MA, U.S.A.). Sections corresponding to − 2 mm from bregma, which contain hippocampus and thalamus, were washed with PBS, incubated with blocking buffer (10% goat serum, 0.3% Trion X-100 in PBS), and then incubated overnight at 4°C with the following primary antibodies: Rabbit anti-Iba1 antibody (1:200) (Wako Pure Chemical, Japan), mouse anti-GFAP antibody-cy3 (1:500) (Millipore Sigma, MO, USA). We used either donkey anti-rabbit IgG-Alexa 594 or 488 (1:200, Thermo Fisher Scientific, Waltham, MA, USA) to recognize rabbit anti-Iba1 antibody. Sections were incubated with DAPI (4′, 6-diamidino-2-phenylindole) to label nuclei. Images were obtained using a Leica TCS SPE confocal system and a Leica DMi8 fluorescence microscope system (Leica Biosystem, IL, USA). Images were captured using a 10X objective. Higher magnification images of selected regions were collected using 20X or 40X objectives. Image analysis was performed using Image J software (National Institutes of Health).

### Lentivirus infection and ROS measurement by flow cytometry.

Control lentivirus (Cx3cr1-IRES-eGFP, initial titer-1.55×10^8^ TU/ml) and Ifi27l2a expressing lentivirus (Cx3cr1-Ifi27l2a-IRES-eGFP, initial titer- 1.07×10^8^ TU/ml) were generated (GeneCopoeia, Rockville, MD, USA) and these virus went through in-house quality control and validation. Sim-A9 cells, a microglia-like cell line, was transduced with control lentivirus (eGFP alone) or Ifi27l2a expressing lentivirus at 2 MOI using polybrene (Millipore Sigma, St. Louise, MO, USA). Five days after infection, cells were incubated with CellRox (Thermo Fisher Scientific, Waltham, MA, USA) for ROS detection or MitoSox (5 *μ*M) (Thermo Fisher Scientific, Waltham, MA, USA) for mitochondrial derived ROS detection for 10 minutes at 37°C. Cells were analyzed using a CytoFLEX S flow cytometer (Beckman Coulter Life Science, Indianapolis, IN, USA). For analysis, a gating strategy was applied first to remove debris using forward (FSC-A) and side scatter (SSC-A). Doublets were also excluded from analysis by FSC-height and width. CellRox Deep Red signal (excitation/emission; 644/665) was collected in the channel (BP 660/20) and the MitoSox Red signal (excitation/emission; 510/580 nm) in the channel (BP 585/42). Data were exported and analyzed with FlowJo software (FlowJo, Tree Star Inc., Ashland, OR, USA). The geometric mean of fluorescence intensities (MFI) and percentage of positive cells were calculated and expressed.

### Mouse Ifi27l2a ELISA.

To check the intracellular levels of Ifi27l2a protein in primary microglia, the murine interferon alpha-inducible protein 27-like protein 2A (Ifi27l2a) was quantified by ELISA following the manufacturer’s recommendations (Abbexa, Cambridge, UK) after washing the cells with PBS two times and collecting lysates in RIPA lysis buffer.

### Real-Time quantitative RT-PCR.

To validate the findings of scRNA-seq data, we performed qRT-PCR. Brains from naive young (3 mons) and aged mice (18–20 mons), or brains from sham and stroked mice (PSD 3 or PSD 14) were harvested and dissected to obtain both cortex and thalamus. For the PLX5672 treatment experiment, the ipsilateral hemisphere was collected instead. Total RNA was purified with TRIzol^™^ Reagent (Thermo Fisher Scientific, Waltham, MA, USA) using the RNeasy Mini Kit (Qiaqen, Germantown, MD, USA) according to the manufacturer’s instructions. Purity of RNA (> 1.7 at 260/280) and concentration of purified RNA were measured by Nano-drop Spectrometer and 1 μg of RNA was used to generate cDNA with iScript^™^ Reverse Transcription Supermix (Bio-Rad, Hercules, CA, USA). The SsoAdvanced Universal SYBR Green Supermix (Bio-Rad, Hercules, CA, USA) was used to detect newly amplified amplicons with a C1000 Touch Thermal Cycler CFX96 Real-Time System (Bio-Rad, Hercules, CA, USA). The PCR cycles were as follows: initial denaturation at 95°C for 30 sec, followed by 40 reaction cycles of 95°C for 5 sec, 56°C for 10 sec, and 72°C for 10 sec. To quantify relative gene expression, we used the ΔΔCt method using Ct values for the gene of interest normalized to GADPH. Data was expressed as fold change relative to control samples. Primer sequences are provided in Extended data Table 1.

### Statistical data analysis.

Statistical data analysis was performed using Prism 7.0.3 (GraphPad Software, San Diego, CA, USA) and R in Rstudio environment with p < 0.05 considered statistically significant. Data are presented as the mean ± standard error of the mean (SEM), and analyzed using an unpaired t-test (for two group comparisons) or a one-way ANOVA with Tukey post-hoc test for multiple comparisons.

## Figures and Tables

**Table 1:** scRNA-seq samples analyzed

	Young		Aged	
Sample	Sham (n = 2)	Stroke (n = 4)	Sham (n = 2)	Stroke (n = 4)
**Name**	AGGR1	AGGR2	AGGR3	AGGR4
**# of cells analyzed**	5706	12866	5174	8226

**Table 2.** Identification of Ifi27l2a as a top gene that is upregulated in MG in aged stroke

Top 5 Genes	Young Stroke (expression level)	Aged Stroke (expression level)	Ave_log FC	Ave_FC
Ifi27l2a	1.10	2.03	0.92	1.90
LgalsS	0.99	1.83	0.84	1.79
Ifitm3	1.15	1.93	0.83	1.78
Lyz2	3.27	3.93	0.65	1.57
Lgals3bp	1.43	2.08	0.65	1.57

**Table 3:** Differential expression of *Ifi27l2a* in each MG sub-clusters in sham and stroke in aged brains

MG sub-clusters	Aged Sham (expression level)	Aged Stroke (expression level)	Average_FC
Homeostatic MG_1- Siglech+	1.419	2.489	1.856
Homeostatic MG_2- P2ry12+	1.279	1.815	1.419
DAM like sub-cluster	**2.762**	**4.910**	**1.777**
MG_Activated_1	1.931	5.251	2.718
MG_Activated_2	2.272	6.276	2.761
MG_Progenitor	3.290	7.628	2.318
